# Body composition after implementation of an enhanced parenteral nutrition protocol in the neonatal intensive care unit: a randomised pilot trial

**DOI:** 10.1080/03014460.2024.2306352

**Published:** 2024-01-31

**Authors:** Emily M. Nagel, Jennifer Super, Nicholas A. Marka, Ellen W Demerath, Sara E Ramel

**Affiliations:** a Division of Epidemiology and Community Health, School of Public Health, University of MN, Minneapolis, MN, USA; b Department of Pediatrics, Division of Neonatology, School of Medicine, University of Minnesota, Minneapolis, MN, USA; c Clinicial and Translational Science Institute, University of Minnesota, Minneapolis, MN, USA

**Keywords:** Prematurity, enhanced nutrition, parenteral nutrition

## Abstract

**Background:**

Very low birthweight (VLBW) infants are at risk for growth failure and poor neurodevelopment. Optimised parenteral nutrition may help promote optimal growth and development, but concerns that provision of enhanced nutrition may contribute to increased early adiposity and later metabolic disease remain.

**Aim:**

To determine associations between provision of an early enhanced parenteral nutrition protocol or standard parenteral nutrition protocol and growth and body composition for VLBW preterm infants in the neonatal intensive care unit.

**Subjects:**

This is a secondary analysis of data from a clinical trial aimed at assessing the feasibility and safety of randomising VLBW preterm infants to Standard (*n* = 45) or Intervention (*n* = 42) parenteral nutrition groups between August 2017 and June 2019.

**Methods:**

We evaluated associations between weekly infant growth and body composition measurements from *n* = 55 infants (Standard = 29, Intervention = 26) that were clinically stable enough to have body composition measurements taken before discharge using mixed effects linear regression models.

**Result:**

No statistically significant associations between nutrition group and infant growth or body composition measures were observed (*p* >.05).

**Conclusion:**

In this pilot trial, enhanced parenteral nutrition in the first week of life was not associated with significant differences in infant growth or body composition during hospitalisation.

## Introduction

The provision of enhanced parenteral nutrition (increased amino acids, lipids, or dextrose) to preterm infants, particularly in the first week of life has been proposed as a clinical tool to offset the harms of inadequate nutrition in the presence of increased needs (Lubchenco et al. 1972; dit Trolli et al. 2012; Lapillonne and Griffin 2013; Uthaya et al. 2016; Alburaki et al. 2021). However, concerns about the feasibility and safety and potential metabolic consequences of implementing aggressive parenteral nutrition protocols combined with a lack of data from randomised controlled trials hinders the regular use of such protocols in preterm infants (Hay 2013). While several retrospective, observational studies have examined associations between early nutritional provision and growth (e.g. weight gain) in preterm infants, healthy preterm infants may receive more nutrition due to medical stability and the ability to tolerate increased volumes in comparison to infants that are critically ill, which may bias the results of observational studies. There is sparse evidence from randomised clinical trials, which help elucidate associations while reducing bias, on the effects of implementing enhanced parenteral nutrition on the quality of weight gain (e.g. body composition parameters) (Wilson et al. 1997; Pappoe et al. 2009; Antonio Costa-Orvay et al. 2011; Can et al. 2012; Meyers et al. 2013; Alja’nini et al. 2021). Assessment of infant weight quality using body composition methodology provides an estimation of fat-mass (FM) versus fat-free mass (FFM), the latter of which is considered an index for brain growth (Skullerud 1985; Binder et al. 2021) and has been linked to improved neurodevelopmental outcomes at 1-year corrected age (Ramel et al. 2016).

The results of our recent, randomised trial revealed that provision of an enhanced parenteral nutrition protocol to preterm infants was not associated with differences in days of hyperglycaemia, hyperbilirubinemia, or hypertriglyceridaemia or differences in the proportion of bronchopulmonary dysplasia, necrotising enterocolitis, and death compared to infants receiving standard parenteral nutrition (Nagel et al. 2023). Thus, the purpose of this secondary analysis was to assess associations between nutrition group (Intervention vs Standard parenteral nutrition group) and preterm infant growth and body composition in the neonatal intensive care unit (NICU).

## Subjects and methods

### Study cohort

Inclusion and exclusion criteria for the study have been described elsewhere (Nagel et al. 2023). Briefly, *n* = 90 early preterm infants born VLBW (very low birth weight) were recruited from the NICU at the University of Minnesota Masonic Children’s Hospital between August 2017 and June 2019. Preterm infants born between 22 + 0 and 31 + 6 weeks’ gestational age and weighing less than 1500 grams at birth were eligible for the study. Exclusion criteria included infant prenatal diagnosis of a clinical condition known to affect adiposity, growth rate, or neurodevelopment. Preterm infants with severe birth asphyxia, or that were enrolled in another study affecting nutritional management, were likely to transfer out of the NICU, and/or with inability to follow up for discharge visits were also excluded from the study. Infants that met the inclusion and exclusion criteria and for which the study team was able to obtain informed consent of parents/guardians within 12 h of birth were enrolled. The final analytical data set for the present study included *n* = 55 infants with complete data available. This study was approved by the University of Minnesota Institutional Review Board (#00000063) and registered at the US National Institutes of Health (ClinicalTrials.gov) #NCT03238768.

### Randomisation and masking

Infants enrolled in the study were stratified by gestational age group (22–25 weeks, 26–29 weeks, or 30–32 weeks at birth). Within each stratum, permuted block randomisation was used to assign infants to either an enhanced parenteral nutrition protocol (Intervention group) or a standard parenteral nutrition protocol (Standard group) while preventing study personnel from being able to predict treatment allocation. The principal investigator, inpatient study coordinator, dietitian, and data analyst were unblinded to each infant’s randomisation group, while other study personnel and parents were blinded to randomisation group. The study coordinator/nurse facilitated randomisation and thus was aware of each infant’s randomisation group. The dietitian was collecting data on the daily nutritional intake and assisting the team in following the protocol but did not share this data with parents/bedside nurses and was not involved in outcome measurement.

### Intervention

**Figure 1.**
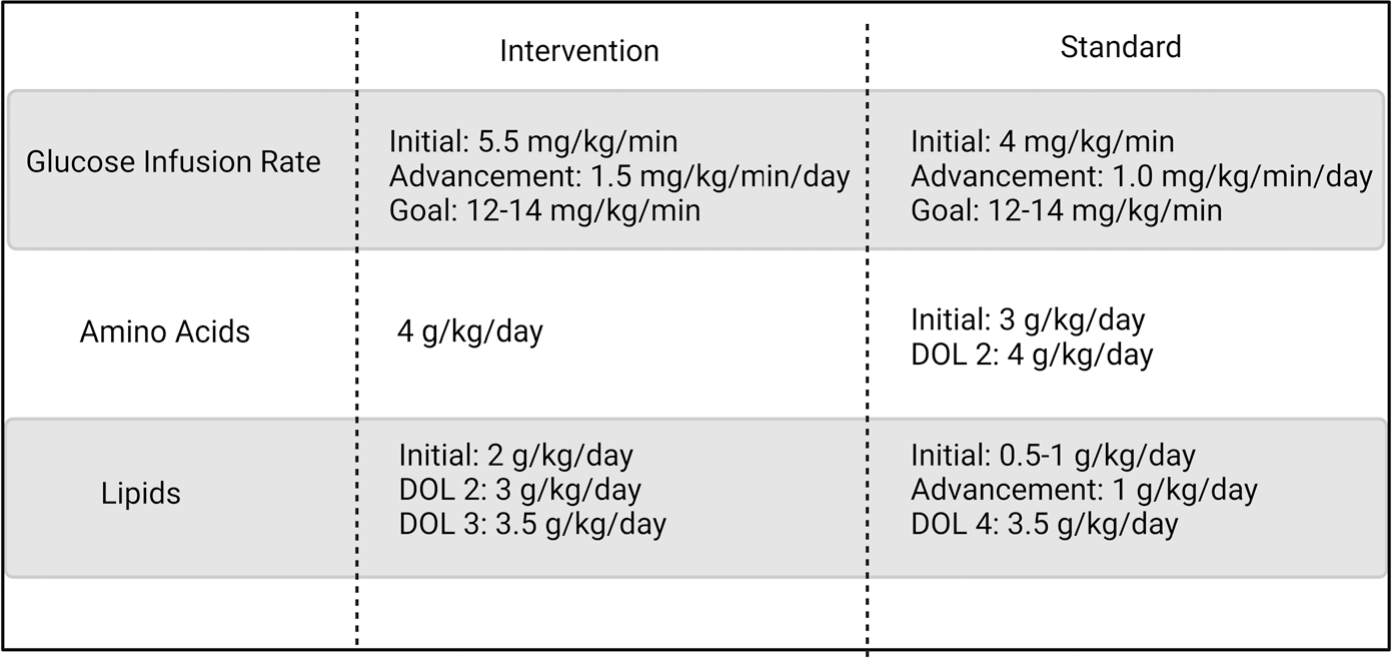
Nutrition protocol for Intervention and Standard Nutrition groups.

The Intervention Group received starter parenteral nutrition (PN) at 80 mL/kg/day, which consisted of 4 g/kg protein and a glucose infusion rate (GIR) of 5.5 mg/kg/min. GIR was advanced by 1.5 mg/kg/min daily until reaching a goal of 12–14 mg/kg/min (Figure 1). Intravenous lipids (100% soybean oil) (IL) were initiated at 2 g/kg/day and increased to 3 g/kg/day on day of life (DOL) 2 and 3.5 g/kg/day on DOL 3. The Standard group received starter PN at 60 mL/kg/day, with 3 g/kg/day protein, GIR of 4 mg/kg/min, and IL of 0.5–1 g/kg/day. GIR was advanced by 1 mg/kg/min daily until the goal of 12–14 mg/kg/min was achieved, and IL were advanced by 1 g/kg/day until the goal of 3.5 g/kg/day was achieved per standard NICU protocol. Protein was advanced to 4 g/kg/day on DOL 2 and maintained at this dose until adequate feeding volumes were achieved. PN goals for both groups were maintained until 40 mL/kg enteral feeds were established and then were decreased proportionally with enteral nutrition intake. Enteral feeds were provided and advanced at the discretion of the clinical team and not dictated by study protocol. In general, feeds were initiated and advanced by 20 mL/kg/day to a goal of 150–160 mL/kg. After the first week of life, the calorie provision from enteral and parenteral nutrition was adjusted to meet goals of 120 kcal/kg and 4 g/kg protein. Most infants received human milk fortified with a bovine-based human milk fortifier and additional protein. Calorie and protein provision of PN were adjusted as enteral feeds were advanced. All PN was administered *via* a central line.

### Body composition and anthropometric measurements

Anthropometric (infant weight in kg and length and head circumference (OFC) in cm) measurements were obtained weekly. The Fenton preterm growth charts were used to calculate z-scores for weight, length, and OFC (Fenton and Kim 2013). Body composition (FM, FFM, %BF (percent body fat)) measurements were conducted weekly using air displacement plethysmography (Pea Pod device; Cosmed Ltd., Concord, California) (Sainz and Urlando 2003; Urlando et al. 2003; Ma et al. 2004; Forsum et al. 2016) beginning when infants were medically stable (able to be without respiratory support for approximately 5 min, central line removed). All measurements were obtained by a trained paediatric research team. Body composition z-scores were calculated using the postconceptional age charts by Norris and colleagues (Norris et al. 2019).

### Feeding data

Detailed nutrient intake data were collected daily by the NICU dietitian until 42 weeks postmenstrual age or until discharge, whichever came first. Data collected included total calories (kcal/kg/day), glucose infusion rate (mg/kg/min), protein (g/kg/day), and lipids (g/kg/day). During the first week of life, birth weight was used for all calculations. After the first week, daily weight was used for calculations or a dosing weight (if one had been determined by the medical team). Due to variable time of birth and subsequent variation in nutrition received, the infant’s first week of nutritional intake was defined as the total number of calories and protein received on days of life (DOL) 2–8. Additionally, deficits in protein (protein g/kg over length of stay-(4 g/kg*length of stay)) and calorie (kcal/kg over length of stay-(120 kcal/kg*length of stay)) over the total length of stay were calculated. These estimated calorie (120 kcal/kg) and protein goals (4 g/kg) are based upon recommendations by the American Dietetic Association (Academy of Nutrition and Dietetics Pediatric Nutrition Practice Group 2016), Association of Parenteral and Enteral Nutrition (American Society for Parenteral and Enteral Nutrition 2019), and the European Society for Parenteral and Enteral Nutrition (Joosten et al. 2018) and reflect the standard of care in our NICU at the time the study was conducted.

### Statistical analyses

This study is a secondary analysis of data from a pilot trial aimed at assessing feasibility/safety of randomising VLBW early preterm infants to an enhanced vs standard parenteral nutrition protocol in the first week of life. For this study, a sample size of 40 infants in each group (80 total) was selected to allow for up to a ~12% loss to follow-up after discharge, which meets the threshold for a sufficiently precise estimate of the variance of body composition and anthropometric z-scores in this population to use in future clinical studies (Julious 2005). Previously, we showed a significant association of early nutrition (protein and calories in first week of life) with FFM gains throughout hospitalisation (*p* <.01 for both) in 34 VLBW preterm infants (Ramel et al. 2016). Thus, we expected that this pilot study, with a sample size 2–3 times larger than used in our previous studies, would be sufficient to detect statistically significant associations. Our primary outcomes of interest were longitudinal measures of infant anthropometrics and body composition, specifically infant length and FFM. Other longitudinal measures of growth (weight and OFC) and body composition, (FM and %BF) were assessed as secondary outcomes. Data were analysed according to a per protocol analysis as not all participants underwent body composition assessment largely due to clinical instability (body composition measurements *via* Pea Pod require infants to be without a central line and independent of respiratory support for at least 5 min).

Differences in total calorie and protein intake over the first week of life (total intake from enteral and parenteral nutrition combined) between groups and protein and calorie deficits during each infant’s length of stay, as well as parenteral nutrition duration (calculated as last age in days at which infant received PN) were evaluated using Wilcoxon tests for data with non-normal distributions due to the smaller sample sizes within groups. Linear mixed effects regression models with random intercepts for infants were used to evaluate associations between nutrition group (Intervention vs Standard) and body composition (FM, FFM, %BF and z-scores) or anthropometric measurements (weight, length, OFC and z-scores). Linear mixed effects models were chosen due to their ability to handle variations in the number of data points per individual and variation in the spacing of repeated measurements. We also assessed the associations between calorie (kcal/kg) and protein (g/kg) intake in the first week of life (from both enteral and parenteral nutrition) with body composition and anthropometric z-scores.

Data were examined for statistical outliers which placed undue leverage on the models and differences were reported if observed (none significantly changed model effects) (Sonnberger 1989). SAS Enterprise Guide 7.1 (SAS Institute, Cary, North Carolina) was used to perform statistical analyses. *P*-values for the primary outcomes were compared using the Sidak correction (Šidák 1967).

## Results

### Demographics

**Figure 2.**
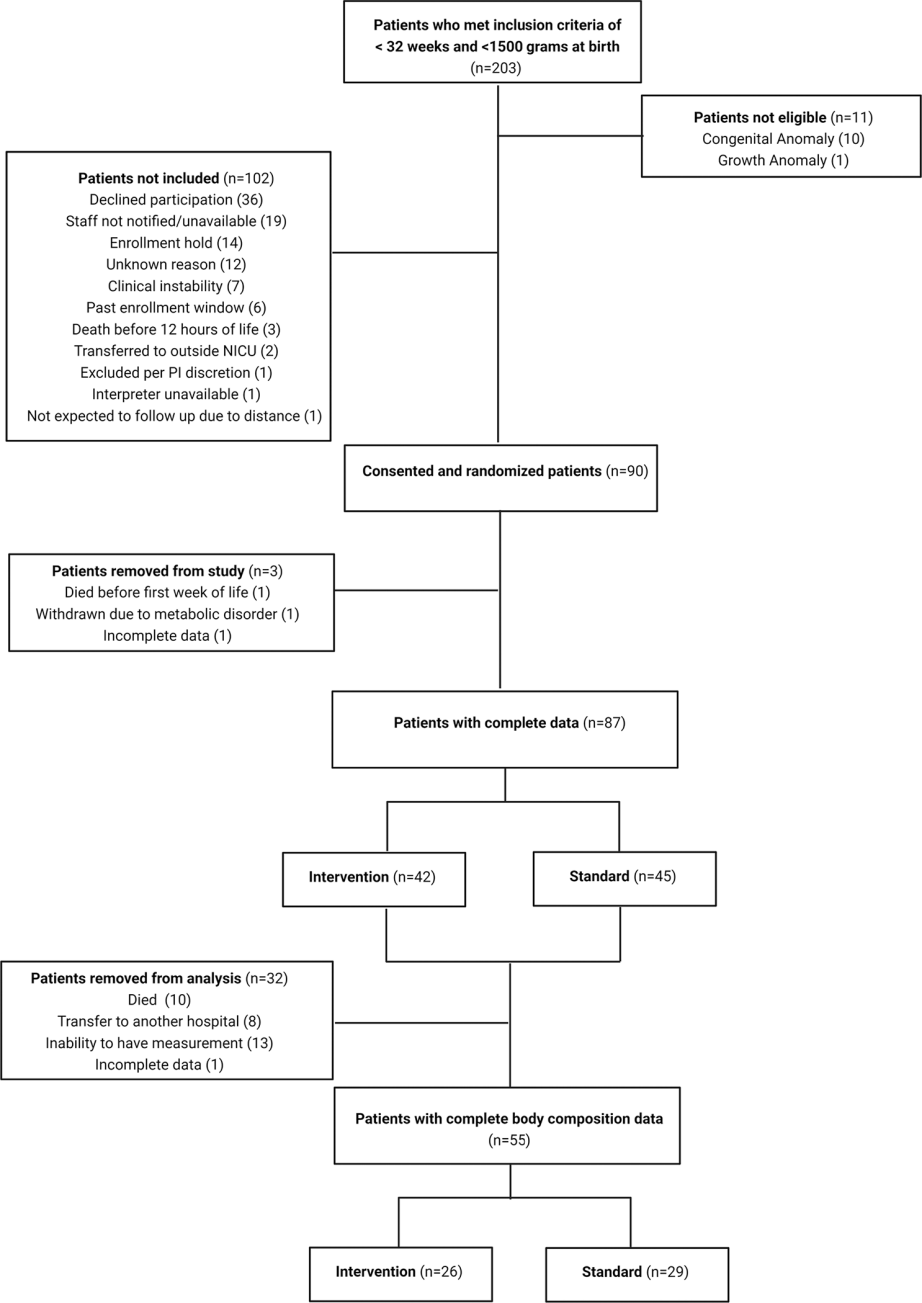
CONSORT diagram for study.

Of the 90 preterm infants enrolled in the study, 55 had body composition and anthropometric measurements available (Intervention group: *n* = 26; Standard group: *n* = 29; Figure 2). The number of available measurements ranged from 1–9 per infant. Characteristics of the study sample are described in Table 1. As expected, due to randomisation, infants in each group had similar descriptive characteristics. Regarding nutritional intake in the first week of life (Table 2), the Intervention group had higher total calorie intake (per kg) than the Standard group (Intervention: median = 105.3, IQR =19.4; Standard: median = 97.0, IQR =17.5). The Intervention group specifically received more calories and protein from parenteral nutrition than the Standard group per protocol. There were no significant differences in total protein intake or in calorie or protein intakes from enteral feeds in the first week of life, as expected per randomisation. Regarding length of stay nutrition, no significant differences between groups in total calorie or protein intake, intake from enteral feedings, or calorie and protein deficits were observed.

### Nutrition and weight quality and growth parameters

There were no significant associations between nutrition group and FFM, FM, %BF or their z-scores (all *p*>.05) (Tables 3 and 4). Similarly, we did not observe significant associations between nutrition group and infant weight, length, OFC or their z-scores (Tables 5 and 6).

We did not observe any significant associations between calorie and protein intakes in the first week of life and infant body composition z-scores during hospitalisation (Table S1), but the directions of the associations were similar to our previous findings (Ramel et al. 2016). Mean calorie intake in the first week of life was not significantly associated with weight or length z-scores, but mean protein intake was positively associated with length z-score (β: 0.98 ± 0.47; *p* =.04).

## Discussion

While several observational studies have examined associations between parenteral nutrition and infant growth outcomes (Wilson et al. 1997; Pappoe et al. 2009; Antonio Costa-Orvay et al. 2011; Can et al. 2012; Meyers et al. 2013; Alja’nini et al. 2021), a lack of evidence from randomised trials, which help clarify outcomes confounded by uncontrolled variables, prevents routine use of enhanced parenteral nutrition for preterm infants at many centres. Thus, reporting the results of such randomised trials is important to innovating nutritional care for preterm infants. Substantial evidence exists for the role of early nutrition and later outcomes for preterm infants, such as neurodevelopment (Lubchenco et al. 1972; Brandt et al. 2003; Stephens et al. 2009; dit Trolli et al. 2012; Lapillonne and Griffin 2013). While early preterm infants have greater adiposity and lower FFM than their term-born peers at term corrected age (Roggero et al. 2009; Ramel et al. 2012), findings from our previous work and others suggest that these differences in body composition resolve in late infancy and the resolution continues into adulthood (Fewtrell et al. 2004; Giannì et al. 2008; Ramel et al. 2011; Griffin and Cooke 2012). It is possible, however, that early body composition differences (i.e. those during hospitalisation) may be linked to later metabolic outcomes, but these relationships have not been thoroughly examined. In this pilot study, we examined for the first time whether randomisation of preterm infants to an enhanced parenteral nutrition protocol in the first week of life was associated with growth and body composition parameters.

Clinicians frequently cite concerns for adverse metabolic outcomes as rationale for not providing preterm infants with enhanced or aggressive parenteral nutrition (Repa et al. 2016; Zamir et al. 2018). However, our study, as a randomised trial vs observational study, adds to the existing body of literature showing that enhanced parenteral nutrition is not associated with significantly increased adiposity in early preterm infants (Antonio Costa-Orvay et al. 2011; Ramel et al. 2011). Further, the feasibility and safety data from this clinical trial suggests that enhanced parenteral nutrition can be provided to early preterm infants without increasing rates of poor clinical outcomes, such as hyperglycaemia, hyperbilirubinemia, or cholestasis (Nagel et al. 2023). Since our study was conducted, published guidelines containing parenteral recommendations for preterm infants advised against provision of an initial dose of amino acids > 3 g/kg/day due to a single trial with an increased rate of sepsis in VLBW preterm infants randomised to an initial dose of 3.5 g/kg/day. (Moltu et al. 2013; Robinson et al. 2023). Although we did not see a difference in positive cultures between our groups (unpublished data) and we did not have significantly different overall protein intake between groups, caution is warranted in providing amino acids beyond 3 g/kg/day.

While, to our knowledge, our study is the first randomised trial of enhanced parenteral nutrition (dextrose and lipids) and body composition outcomes in preterm infants, other studies have examined augmentation of specific macronutrients in parenteral nutrition. Uthaya et al. conducted a randomised, double blind controlled trial of immediate vs incremental delivery of amino acids in parenteral nutrition and use of SMOF-lipids vs standard lipids (Uthaya et al. 2016). Infant adiposity (*via* MRI) was a primary outcome of interest among other clinical outcomes at term age in 133 preterm infants. The incremental group received amino acids starting at 1.7 g/kg on DOL 1 and increasing to 2.1 g/kg on DOL 2 to a maximum of 2.7 g/kg on DOL 3. The immediate group received 3.6 g/kg of amino acids from DOL 1 onward. Lipids (either mixed lipids containing soybean oil, medium chain triglycerides, olive oil, and fish oil or soy-based lipids) were provided at 2 g/kg on DOL 1 and increased to 3 g/kg from DOL 2 onward. No significant differences in adiposity were observed among groups. Although the nutrition protocol and body composition methods utilised were different, implementation of an enhanced parenteral nutrition protocol in this study of a larger group of preterm infants yielded similar results to our study.

Another randomised controlled trial by Alburaki et al. assessed the administration of a higher dose of early parenteral soy-based lipids in the first week of life in a sample of 83 very low birth weight preterm infants (Alburaki et al. 2021). In the intervention group, lipids were initiated at 2 g/kg/day and increased to 3 g/kg the following day. Infants in the control group received a starting lipid dose of 0.5–1 g/kg/day, which was increased daily by 0.5–1 g/kg/day until reaching 3 g/kg/day of lipids. Infants in the intervention group had a lower percent weight loss and lower incidence of extrauterine growth restriction at 36 weeks postmenstrual age. The rate of hypertriglyceridaemia was similar between groups. Although the authors did not assess body composition, the growth parameters examined were improved by enhanced lipid provision.

While we only observed a small difference in median calories received per weight of approximately 8 kcal/kg between the Intervention and Standard groups (Stephens et al. 2009), our previous studies indicate that nutrition in the first week of life (protein and energy intake) is positively associated with FFM gains but not FM gains throughout hospitalisation (Ramel et al. 2016). Thus, small differences in calorie and protein intake may be important to optimising body composition for preterm infants.

The primary limitation of our study was the number of infants who had body composition measurements available. While 87 infants were originally randomised to nutrition groups, 32 of those infants did not have body composition measurements taken, mostly due to clinical instability. Approximately 47% of the missing data was from infants in the Intervention group and 53% from infants in the Standard nutrition group. Infants that did not have measurements taken had a lower mean gestational age at birth than those infants with available measurements (Mean, SD: 26.4 weeks, 3.04 vs 27.6, weeks 2.01). Thus, our results should be interpreted with caution for preterm infants < 27 weeks of age at birth. However, the number of infants in the final analytic sample (*n* = 55) is ~62% larger than the number in our previous observational study (*n* = 34 VLBW preterm infants), which found significant associations between early nutrition (protein and calories in first week of life) and FFM gains throughout hospitalisation (Ramel et al. 2016). Further, the data from the current study are longitudinal and represent over *n* = 150 body composition measurements taken throughout hospitalisation. Finally, the number of infants in each group (Intervention: *n* = 26; Standard: *n* = 29) meets the threshold for a sufficiently precise estimate of the variance of body composition and anthropometric z-scores in this population and can be used to estimate the sample size needed for future clinical studies. We also acknowledge that our nutrition intervention was limited to the first week of life; thus, we did not collect data on human milk vs donor milk or infant formula intake or directly measure the protein content of human milk in this study. While higher intake of human milk can affect body composition (Piemontese et al. 2018), our NICU has a high utilisation of human milk, with ~70% of infants receiving human milk through discharge. Thus, we would expect groups to contain similar numbers of infants receiving human milk due to randomisation.

Due to the results of our previous study, we expected preterm infants in the Intervention group to have greater FFM than infants in the Standard nutrition group. However, we did not see the same association in the current study. Although the Intervention group received significantly more calories (per kg) in the first week of life, it is possible that the difference in calories was not sufficient to cause changes in body composition. Overall, larger randomised trials of enhanced parenteral nutrition in preterm infants are needed to examine these relationships more closely. Despite these limitations, we were able to successfully implement an enhanced parenteral nutrition protocol in a cohort of early preterm infants and collect detailed clinical nutritional and growth quality data throughout each infant’s hospital stay.

In this pilot trial, we found no meaningful associations between infant anthropometric or body composition parameters and randomisation to an enhanced nutrition protocol vs standard protocol in the first week of life. Further work is needed to confirm our findings in a larger cohort of infants and to determine if provision of early enhanced parenteral nutrition is associated with later growth and body composition outcomes.

## Supplementary Material

Supplemental materials

## Figures and Tables

**Table 1.** Descriptive characteristics of preterm infants (*n* = 55)^a^.

Variable	Intervention Group (*n* = 26)	Standard Group (*n* = 29)

Gestational Age at Birth (weeks)	27.1 ± 2.18	28.0 ± 1.99
Birth Weight (g)	919 ± 243	1051 ± 241
Birth Weight z-score	−0.16 ± 0.83	−0.04 ± 0.83
SGA at Birth^b^	4 (15%)	2 (7%)
Birth Length (cm)	34.6 ± 3.24	35.8 ± 3.14
Birth Length z-score	−0.11 ± 0.98	−0.11 ± 0.93
Birth OFC (cm)	23.7 ± 2.80	25.0 ± 3.82
Birth OFC z-score	−0.52 ± 1.84	−0.33 ± 2.04
Sex (male)	15 (52%)	13 (50%)
Race		
White	15 (65%)	21 (78%)
Asian	3 (13%)	3 (11%)
Black	5 (22%)	2 (7%)
Other	0	1 (4%)
Ethnicity		
Hispanic/Latino	2 (9%)	2 (9%)
Not Hispanic/Latino	17 (77%)	25 (89%)
Not reported	3 (14%)	1 (4%)
Length of Stay (days)	81.5 (47)	81 (44)
Discharge Body		
Composition^c^		
PMA at Discharge	39.4 ± 4.09	39.1 ± 3.89
Measurement (weeks)		
Percent Body Fat (%)	17.7 (10.5)	17.3 (7.80)
Percent Body Fat	1.74 (2.74)	1.96 (2.27)
z-score		
Fat Mass (kg)	0.52 (0.47)	0.60 (0.33)
Fat Mass z-score	1.09 (2.13)	1.80 (2.14)
Fat-Free Mass (kg)	2.46 (0.69)	2.51 (0.54)
Fat-Free Mass z-score	−0.72 (2.30)	−0.90 (1.63)

a Continuous variables expressed as mean ± standard deviation, median (interquartile range), and categorical variables as n (percentage).

b SGA defined as <10%tile weight for gestational age at birth.

c Body composition z-scores calculated using postconceptional charts from Norris et al. (Norris et al. 2019)(Norris et al. 2019)(Norris et al. 2019)(Norris et al. 2019)(Norris et al. 2019).

OFC, head circumference; PMA, postmenstrual age; SGA, small for gestational age.

**Table 2.** Nutritional intake by study group (*n* = 55).

Variable	Intervention Group (*n* = 26)	Standard Group (*n* = 29)	
			
First week of life nutrition^a^	Data Value^b^	Data Value^b^	*p*-value^c^

Total calorie intake (kcal/kg)	105.3 (19.4)	97.0 (17.5)	0.03
Total protein intake (g/kg)	4.12 (0.34)	4.19 (0.52)	0.59
Calories from parenteral nutrition (kcal/kg)	83.6 (13.7)	67.7 (17.7)	<.0001
Protein from parenteral nutrition (g/kg)	3.78 (0.36)	3.54 (0.74)	0.04
Calories from enteral nutrition (kcal/kg)	16.9 (34.8)	30.6 (37.8)	0.25
Protein from enteral nutrition (g/kg)	0.35 (0.62)	0.71 (1.04)	0.27

Length of stay nutrition	Data Value^b^	Data Value^b^	

Total Calorie intake (kcal/kg)	118.6 (7.46)	117.2 (7.11)	0.13
Total Protein intake (g/kg)	4.08 (0.18)	4.15 (0.17)	0.62
Calories from parenteral nutrition (kcal/kg)	17.1 (9.84)	10.6 (11.0)	0.04
Protein from parenteral nutrition (g/kg)	0.69 (0.40)	0.51 (0.34)	0.12
Parenteral nutrition duration (days)	32.6 (33)	18.0 (42)	0.39
Calories from enteral nutrition (kcal/kg)	104.4 (15.7)	109.5 (9.95)	0.42
Protein from enteral nutrition (g/kg)	3.47 (0.45)	3.64 (0.43)	0.14
Calorie deficit	43.9 (558.6)	−88.1 (537.4)	0.20
Protein deficit	8.17 (11.8)	12.3 (14.5)	0.52

a Calculated as DOL 2–8.

b Data are median (Interquartile Range).

c Differences between groups evaluated using Wilcoxon tests.

d Calculated as the last age in days at which infant received PN.

Calorie deficit = total calories − (120 kcal/kg*length of stay); a negative value indicates total calorie intake was less than goal of 120 kcal/kg/day.

Protein deficit = total protein − (4 g/kg*length of stay); a negative value indicates total protein intake was less than goal of 4 g/kg/day.

**Table 3.** Association of nutrition group and body composition over length of stay (*n* = 55).

Nutrition Group	Fat-free Mass (kg)		Fat-free Mass (z-scores)	
		
	β ± SE	*p*-value	β ± SE	*p*-value

Intervention	−0.02 ± 0.14	0.90	−0.26 ± 0.33	0.44
Standard	Ref.	---	Ref.	---

	Fat Mass (kg)		Fat Mass (z-scores)	

	β ± SE	*p*-value	β ± SE	*p*-value

Intervention	0.04 ± 0.09	0.68	0.23 ± 0.45	0.61
Standard	Ref.	---	Ref.	---

	Percent Body Fat (%)		Percent Body Fat (z-scores)	

	β ± SE	*p*-value	β ± SE	*p*-value

Intervention	1.10 ± 1.80	0.55	0.24 ± 0.46	0.60
Standard	Ref.	---	Ref.	---

**Table 4.** Association of nutrition group and body composition over length of stay (*n* = 55) adjusted for SGA status and birthweight.

Nutrition Group	Fat-free Mass (kg)		Fat-free Mass (z-scores)	
		
	β ± SE	*p*-value	β ± SE	*p*-value

Intervention	−0.08 ± 0.14	0.57	−0.08 ± 0.21	0.70
Standard	Ref.	---	Ref.	---

	Fat Mass (kg)		Fat Mass (z-scores)	

	β ± SE	*p*-value	β ± SE	*p*-value

Intervention	−0.03 ± 0.08	0.68	0.37 ± 0.44	0.41
Standard	Ref.	---	Ref.	---

	Percent Body Fat (%)		Percent Body Fat (z-scores)	

	β ± SE	*p*-value	β ± SE	*p*-value

Intervention	−0.44 ± 1.65	0.79	0.005 ± 0.44	0.99
Standard	Ref.	---	Ref.	---

**Table 5.** Association of nutrition group and anthropometric measurements over length of stay (*n* = 55).

Nutrition Group	Weight (kg)		Weight (z-scores)^a^	
		
	β ± SE	*p*-value	β ± SE	*p*-value

Intervention	0.03 ± 0.21	0.88	−0.12 ± 0.25	0.62
Standard	Ref.	---	Ref.	---

	Length (cm)		Length (z-scores)^a^	

	β ± SE	*p*-value	β ± SE	*p*-value

Intervention	0.03 ± 0.86	0.97	−0.19 ± 0.26	0.47
Standard	Ref.	---	Ref.	---

	OFC (cm)		OFC (z-scores)	

	β ± SE	*p*-value	β ± SE	*p*-value

Intervention	−0.50 ± 0.57	0.40	−0.45 ± 0.23	0.052
Standard				

a Fenton growth charts used to calculate weight and length z-scores; *n* = 53 for z-scores.

**Table 6.** Association of nutrition group and anthropometric measurements over length of stay (adjusted for SGA status and birthweight) (*n* = 55).

Nutrition Group	Weight (kg)		Weight (z-scores)^a^	
		
	β ± SE	*p*-value	β ± SE	*p*-value

Intervention	−0.10 ± 0.20	0.63	−0.01 ± 0.17	0.96
Standard	Ref.	---	Ref.	---

	Length (cm)		Length (z-scores)^a^	

	β ± SE	*p*-value	β ± SE	*p*-value

Intervention	0.23 ± 0.88	0.80	−0.10 ± 0.23	0.66
Standard	Ref.	---	Ref.	---

	OFC (cm)		OFC (z-scores)	

	β ± SE	P-value	β ± SE	P-value

Intervention	−0.44 ± 0.58	0.45	−0.38 ± 0.21	0.07
Standard				

a Fenton growth charts used to calculate weight and length z-scores; *n* = 53 for z-scores

Weights: Adjusted for SGA status and birthweight (kg); weight z-score adjusted for SGA status and birthweight z-score

Length and OFC: adjusted for SGA status

## Data Availability

Deidentified data used in this manuscript are available upon request.
