# Supplementary material for: Body composition after implementation of an enhanced parenteral nutrition protocol in the neonatal intensive care unit: a randomised pilot trial
Source: Ann Hum Biol. Author manuscript; Available in PMC 2025 Feb 1. (PMC10964316; doi:10.1080/03014460.2024.2306352)
Supplement: Supplemental materials [file NIHMS1973327-supplement-Supplemental_materials.pdf]

SUPPLEMENTAL DATA

Supplemental Table 1. Association of calorie and protein intakes in the first week of life with infant body composition z-scores during hospitalization (n=53)

Variable	**FFM (z-scores)**	
	Crude	
	β ± SE	P-value
Mean kcal/kg	0.01 ± 0.01	0.31
Mean pro/kg	0.44 ± 0.37	0.93
	**Fat Mass (z-scores)**	
	Crude	
	β ± SE	P-value
Mean kcal/kg	-0.003 ± 0.02	0.87
Mean pro/kg	0.27 ± 0.54	0.62
	**Percent Body Fat (z-scores)**	
	Crude	
	β ± SE	P-value
Mean kcal/kg	-0.01 ± 0.02	0.50
Mean pro/kg	0.07 ± 0.54	0.89

Supplemental Table 2. Association of calorie and protein intakes in the first week of life with infant weight and length z-scores during hospitalization (n=53)

Variable	**Weight (z-scores)**	
	β ± SE	P-value
Mean kcal/kg	0.004 ± 0.01	0.72
Mean pro/kg	0.19 ± 0.46	0.68
	**Length (z-scores)**	
	β ± SE	P-value
Mean kcal/kg	0.02 ± 0.01	0.10
Mean pro/kg	0.98 ± 0.47	**0.04**
	**OFC (z-scores)**	
Mean kcal/kg	0.016 ± 0.01	0.08
Mean pro/kg	0.63 ± 0.44	0.16

Supplemental Table 3. Data of infants who did not have body composition measurements

Variable	Missing Body Composition (n=32)
Group	
Intervention	15 (47)
Standard	17 (53)
Gestational age at birth (weeks)	26.4 ±3.04
Sex (male)	17 (53)
